# Supplementary material for: Irritable Bowel Syndrome with Diarrhea (IBS-D): Effects of Clostridium butyricum CBM588 Probiotic on Gastrointestinal Symptoms, Quality of Life, and Gut Microbiota in a Prospective Real-Life Interventional Study
Source: Microorganisms. 2025 May 15;13(5):1139. doi: 10.3390/microorganisms13051139 (PMC12113862; doi:10.3390/microorganisms13051139)
Supplement: Supplementary file 1 [file microorganisms-13-01139-s001.zip › microorganisms-3605196-supplementary.pptx]

## Slide 1
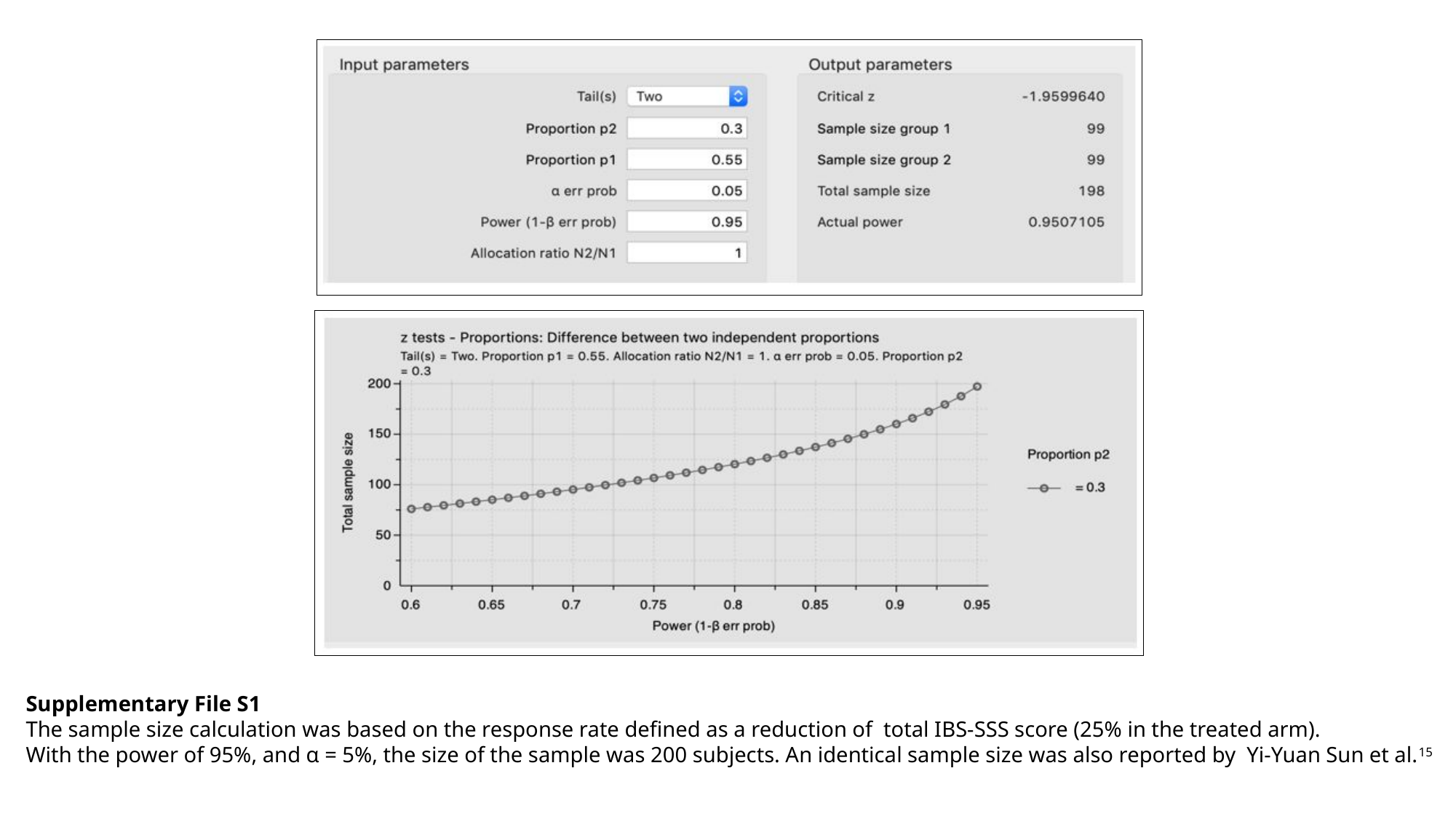

Supplementary File S1
The sample size calculation was based on the response rate defined as a reduction of total IBS-SSS score (25% in the treated arm).
With the power of 95%, and α = 5%, the size of the sample was 200 subjects. An identical sample size was also reported by Yi-Yuan Sun et al.15

## Slide 2
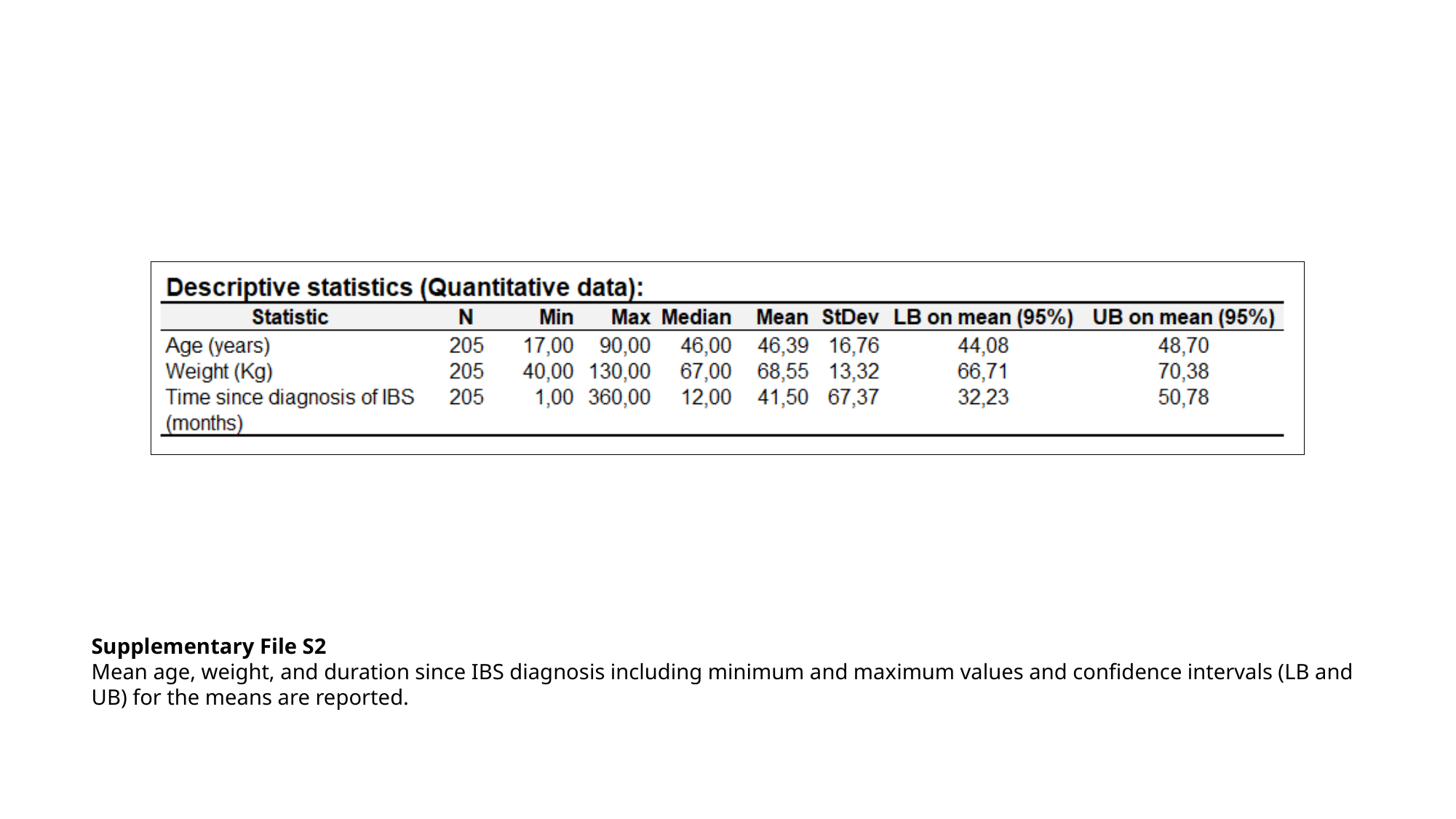

Supplementary File S2
Mean age, weight, and duration since IBS diagnosis including minimum and maximum values and confidence intervals (LB and UB) for the means are reported.

## Slide 3
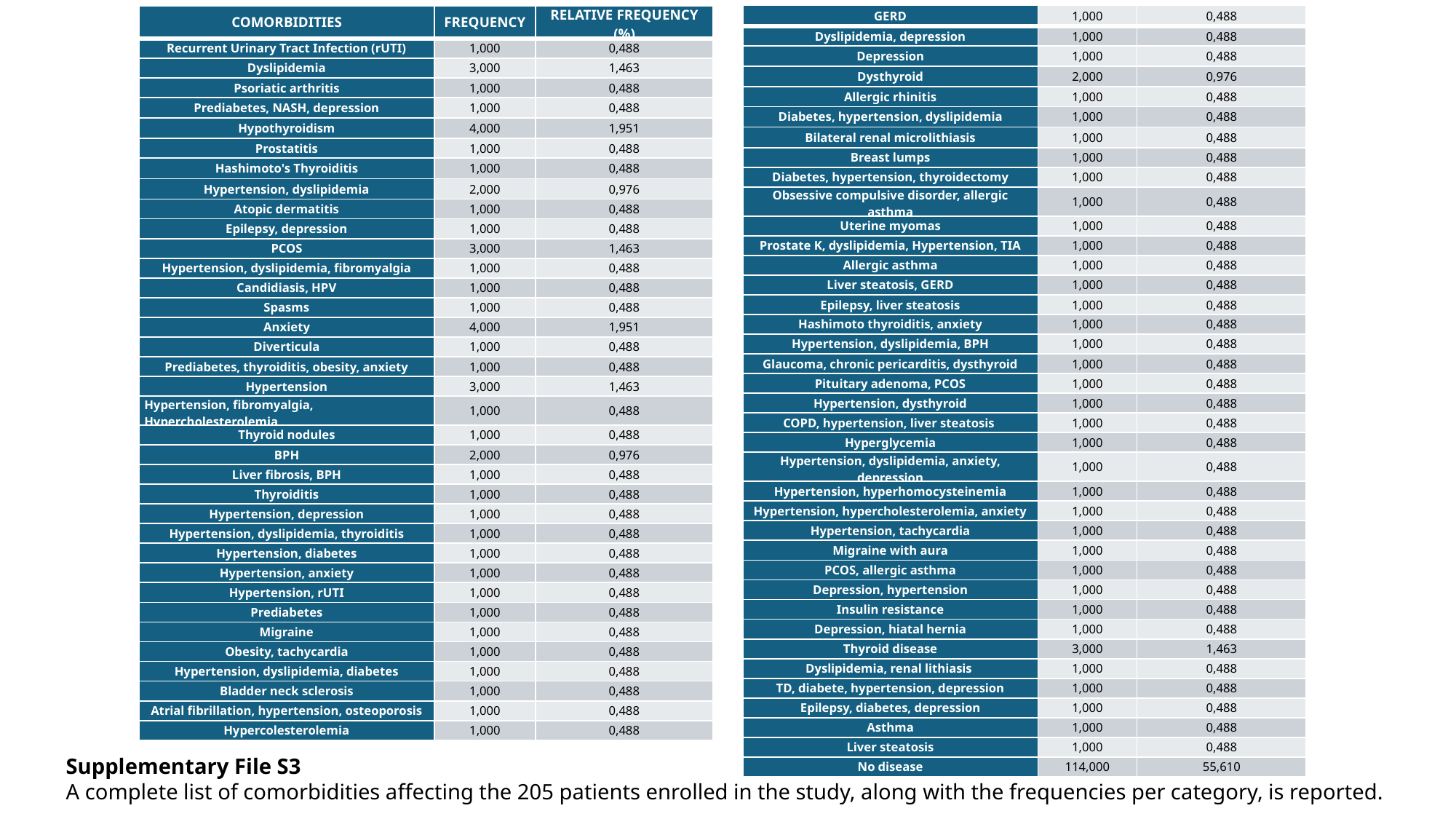

| GERD | 1,000 | 0,488 |
| --- | --- | --- |
| Dyslipidemia, depression | 1,000 | 0,488 |
| Depression | 1,000 | 0,488 |
| Dysthyroid | 2,000 | 0,976 |
| Allergic rhinitis | 1,000 | 0,488 |
| Diabetes, hypertension, dyslipidemia | 1,000 | 0,488 |
| Bilateral renal microlithiasis | 1,000 | 0,488 |
| Breast lumps | 1,000 | 0,488 |
| Diabetes, hypertension, thyroidectomy | 1,000 | 0,488 |
| Obsessive compulsive disorder, allergic asthma | 1,000 | 0,488 |
| Uterine myomas | 1,000 | 0,488 |
| Prostate K, dyslipidemia, Hypertension, TIA | 1,000 | 0,488 |
| Allergic asthma | 1,000 | 0,488 |
| Liver steatosis, GERD | 1,000 | 0,488 |
| Epilepsy, liver steatosis | 1,000 | 0,488 |
| Hashimoto thyroiditis, anxiety | 1,000 | 0,488 |
| Hypertension, dyslipidemia, BPH | 1,000 | 0,488 |
| Glaucoma, chronic pericarditis, dysthyroid | 1,000 | 0,488 |
| Pituitary adenoma, PCOS | 1,000 | 0,488 |
| Hypertension, dysthyroid | 1,000 | 0,488 |
| COPD, hypertension, liver steatosis | 1,000 | 0,488 |
| Hyperglycemia | 1,000 | 0,488 |
| Hypertension, dyslipidemia, anxiety, depression | 1,000 | 0,488 |
| Hypertension, hyperhomocysteinemia | 1,000 | 0,488 |
| Hypertension, hypercholesterolemia, anxiety | 1,000 | 0,488 |
| Hypertension, tachycardia | 1,000 | 0,488 |
| Migraine with aura | 1,000 | 0,488 |
| PCOS, allergic asthma | 1,000 | 0,488 |
| Depression, hypertension | 1,000 | 0,488 |
| Insulin resistance | 1,000 | 0,488 |
| Depression, hiatal hernia | 1,000 | 0,488 |
| Thyroid disease | 3,000 | 1,463 |
| Dyslipidemia, renal lithiasis | 1,000 | 0,488 |
| TD, diabete, hypertension, depression | 1,000 | 0,488 |
| Epilepsy, diabetes, depression | 1,000 | 0,488 |
| Asthma | 1,000 | 0,488 |
| Liver steatosis | 1,000 | 0,488 |
| No disease | 114,000 | 55,610 |
| COMORBIDITIES | FREQUENCY | RELATIVE FREQUENCY (%) |
| --- | --- | --- |
| Recurrent Urinary Tract Infection (rUTI) | 1,000 | 0,488 |
| Dyslipidemia | 3,000 | 1,463 |
| Psoriatic arthritis | 1,000 | 0,488 |
| Prediabetes, NASH, depression | 1,000 | 0,488 |
| Hypothyroidism | 4,000 | 1,951 |
| Prostatitis | 1,000 | 0,488 |
| Hashimoto's Thyroiditis | 1,000 | 0,488 |
| Hypertension, dyslipidemia | 2,000 | 0,976 |
| Atopic dermatitis | 1,000 | 0,488 |
| Epilepsy, depression | 1,000 | 0,488 |
| PCOS | 3,000 | 1,463 |
| Hypertension, dyslipidemia, fibromyalgia | 1,000 | 0,488 |
| Candidiasis, HPV | 1,000 | 0,488 |
| Spasms | 1,000 | 0,488 |
| Anxiety | 4,000 | 1,951 |
| Diverticula | 1,000 | 0,488 |
| Prediabetes, thyroiditis, obesity, anxiety | 1,000 | 0,488 |
| Hypertension | 3,000 | 1,463 |
| Hypertension, fibromyalgia, Hypercholesterolemia | 1,000 | 0,488 |
| Thyroid nodules | 1,000 | 0,488 |
| BPH | 2,000 | 0,976 |
| Liver fibrosis, BPH | 1,000 | 0,488 |
| Thyroiditis | 1,000 | 0,488 |
| Hypertension, depression | 1,000 | 0,488 |
| Hypertension, dyslipidemia, thyroiditis | 1,000 | 0,488 |
| Hypertension, diabetes | 1,000 | 0,488 |
| Hypertension, anxiety | 1,000 | 0,488 |
| Hypertension, rUTI | 1,000 | 0,488 |
| Prediabetes | 1,000 | 0,488 |
| Migraine | 1,000 | 0,488 |
| Obesity, tachycardia | 1,000 | 0,488 |
| Hypertension, dyslipidemia, diabetes | 1,000 | 0,488 |
| Bladder neck sclerosis | 1,000 | 0,488 |
| Atrial fibrillation, hypertension, osteoporosis | 1,000 | 0,488 |
| Hypercolesterolemia | 1,000 | 0,488 |
Supplementary File S3
A complete list of comorbidities affecting the 205 patients enrolled in the study, along with the frequencies per category, is reported.

## Slide 4
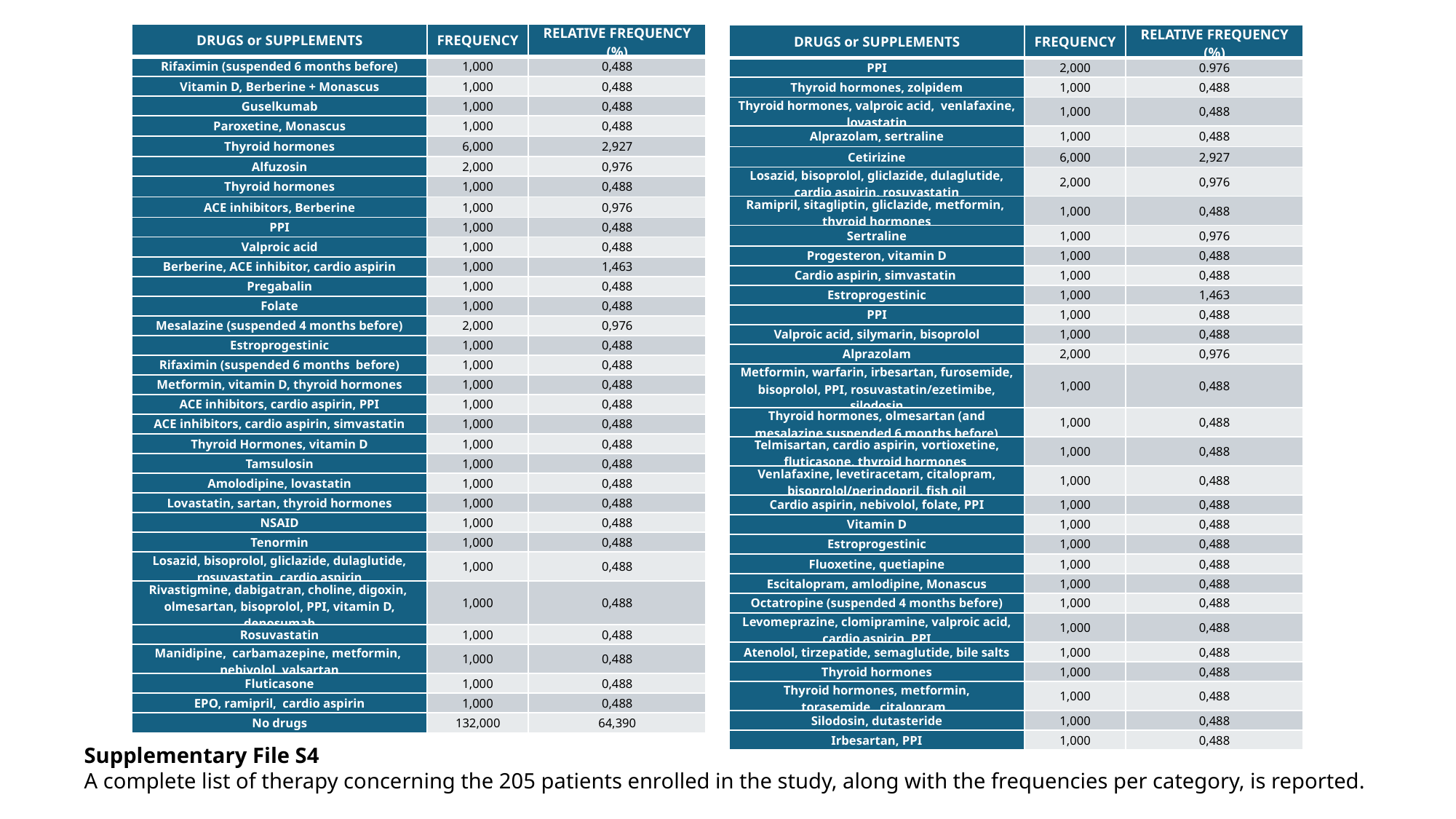

| DRUGS or SUPPLEMENTS | FREQUENCY | RELATIVE FREQUENCY (%) |
| --- | --- | --- |
| Rifaximin (suspended 6 months before) | 1,000 | 0,488 |
| Vitamin D, Berberine + Monascus | 1,000 | 0,488 |
| Guselkumab | 1,000 | 0,488 |
| Paroxetine, Monascus | 1,000 | 0,488 |
| Thyroid hormones | 6,000 | 2,927 |
| Alfuzosin | 2,000 | 0,976 |
| Thyroid hormones | 1,000 | 0,488 |
| ACE inhibitors, Berberine | 1,000 | 0,976 |
| PPI | 1,000 | 0,488 |
| Valproic acid | 1,000 | 0,488 |
| Berberine, ACE inhibitor, cardio aspirin | 1,000 | 1,463 |
| Pregabalin | 1,000 | 0,488 |
| Folate | 1,000 | 0,488 |
| Mesalazine (suspended 4 months before) | 2,000 | 0,976 |
| Estroprogestinic | 1,000 | 0,488 |
| Rifaximin (suspended 6 months before) | 1,000 | 0,488 |
| Metformin, vitamin D, thyroid hormones | 1,000 | 0,488 |
| ACE inhibitors, cardio aspirin, PPI | 1,000 | 0,488 |
| ACE inhibitors, cardio aspirin, simvastatin | 1,000 | 0,488 |
| Thyroid Hormones, vitamin D | 1,000 | 0,488 |
| Tamsulosin | 1,000 | 0,488 |
| Amolodipine, lovastatin | 1,000 | 0,488 |
| Lovastatin, sartan, thyroid hormones | 1,000 | 0,488 |
| NSAID | 1,000 | 0,488 |
| Tenormin | 1,000 | 0,488 |
| Losazid, bisoprolol, gliclazide, dulaglutide, rosuvastatin, cardio aspirin | 1,000 | 0,488 |
| Rivastigmine, dabigatran, choline, digoxin, olmesartan, bisoprolol, PPI, vitamin D, denosumab | 1,000 | 0,488 |
| Rosuvastatin | 1,000 | 0,488 |
| Manidipine, carbamazepine, metformin, nebivolol, valsartan | 1,000 | 0,488 |
| Fluticasone | 1,000 | 0,488 |
| EPO, ramipril, cardio aspirin | 1,000 | 0,488 |
| No drugs | 132,000 | 64,390 |
| DRUGS or SUPPLEMENTS | FREQUENCY | RELATIVE FREQUENCY (%) |
| --- | --- | --- |
| PPI | 2,000 | 0.976 |
| Thyroid hormones, zolpidem | 1,000 | 0,488 |
| Thyroid hormones, valproic acid, venlafaxine, lovastatin | 1,000 | 0,488 |
| Alprazolam, sertraline | 1,000 | 0,488 |
| Cetirizine | 6,000 | 2,927 |
| Losazid, bisoprolol, gliclazide, dulaglutide, cardio aspirin, rosuvastatin | 2,000 | 0,976 |
| Ramipril, sitagliptin, gliclazide, metformin, thyroid hormones | 1,000 | 0,488 |
| Sertraline | 1,000 | 0,976 |
| Progesteron, vitamin D | 1,000 | 0,488 |
| Cardio aspirin, simvastatin | 1,000 | 0,488 |
| Estroprogestinic | 1,000 | 1,463 |
| PPI | 1,000 | 0,488 |
| Valproic acid, silymarin, bisoprolol | 1,000 | 0,488 |
| Alprazolam | 2,000 | 0,976 |
| Metformin, warfarin, irbesartan, furosemide, bisoprolol, PPI, rosuvastatin/ezetimibe, silodosin | 1,000 | 0,488 |
| Thyroid hormones, olmesartan (and mesalazine suspended 6 months before) | 1,000 | 0,488 |
| Telmisartan, cardio aspirin, vortioxetine, fluticasone, thyroid hormones | 1,000 | 0,488 |
| Venlafaxine, levetiracetam, citalopram, bisoprolol/perindopril, fish oil | 1,000 | 0,488 |
| Cardio aspirin, nebivolol, folate, PPI | 1,000 | 0,488 |
| Vitamin D | 1,000 | 0,488 |
| Estroprogestinic | 1,000 | 0,488 |
| Fluoxetine, quetiapine | 1,000 | 0,488 |
| Escitalopram, amlodipine, Monascus | 1,000 | 0,488 |
| Octatropine (suspended 4 months before) | 1,000 | 0,488 |
| Levomeprazine, clomipramine, valproic acid, cardio aspirin, PPI | 1,000 | 0,488 |
| Atenolol, tirzepatide, semaglutide, bile salts | 1,000 | 0,488 |
| Thyroid hormones | 1,000 | 0,488 |
| Thyroid hormones, metformin, torasemide, citalopram, | 1,000 | 0,488 |
| Silodosin, dutasteride | 1,000 | 0,488 |
| Irbesartan, PPI | 1,000 | 0,488 |
Supplementary File S4
A complete list of therapy concerning the 205 patients enrolled in the study, along with the frequencies per category, is reported.

## Slide 5
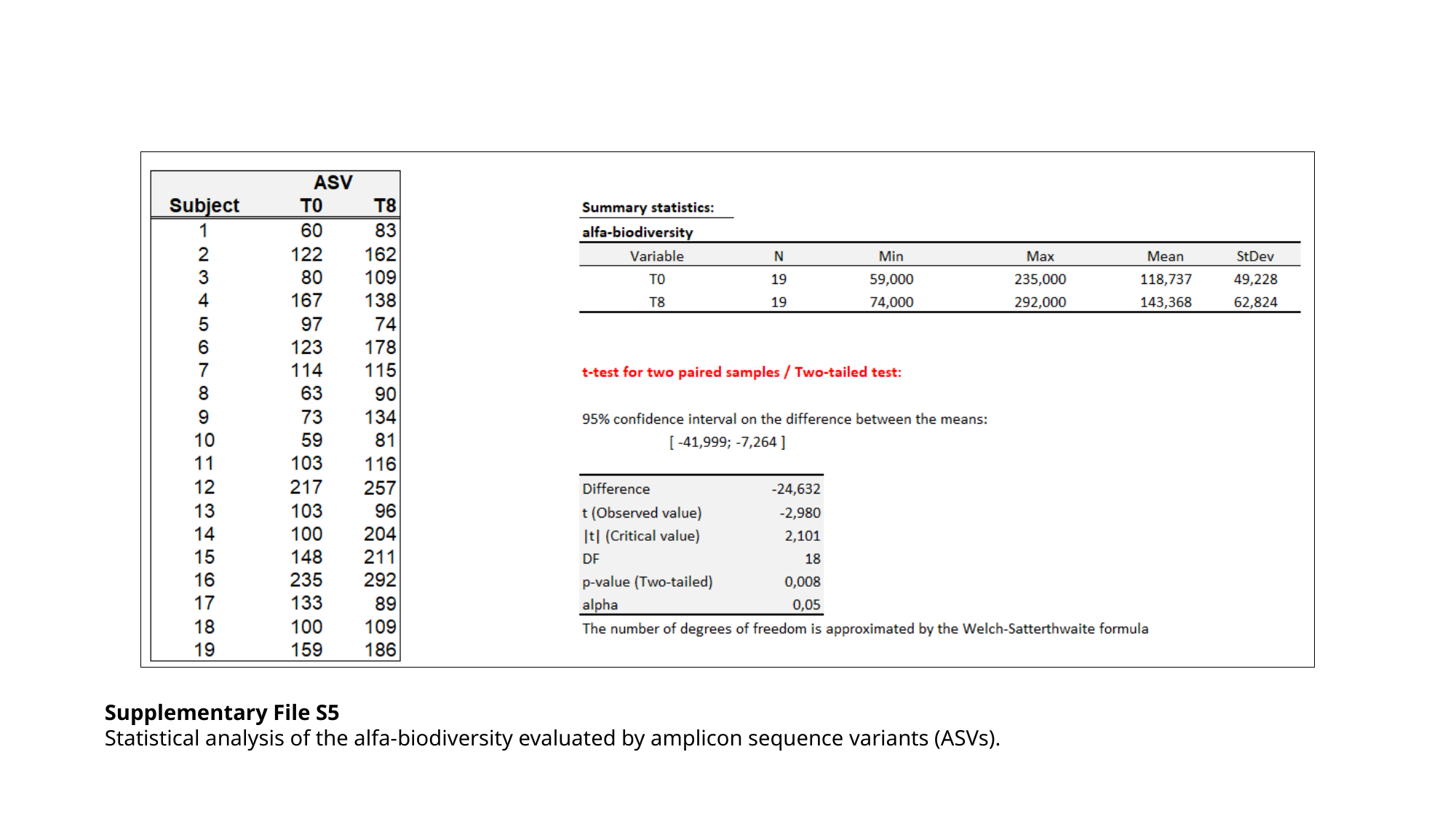

Supplementary File S5
Statistical analysis of the alfa-biodiversity evaluated by amplicon sequence variants (ASVs).

## Slide 6
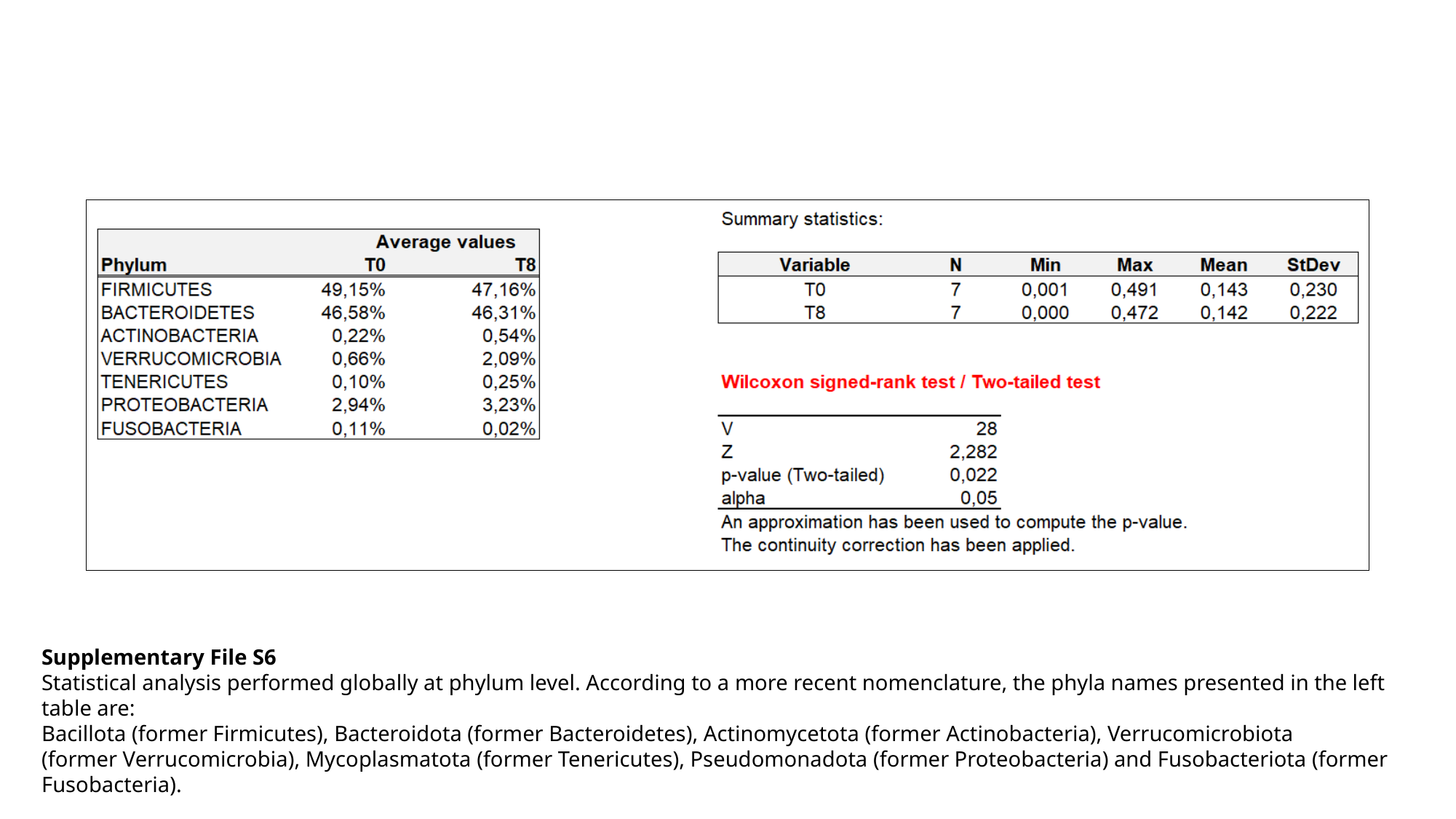

Supplementary File S6
Statistical analysis performed globally at phylum level. According to a more recent nomenclature, the phyla names presented in the left table are:
Bacillota (former Firmicutes), Bacteroidota (former Bacteroidetes), Actinomycetota (former Actinobacteria), Verrucomicrobiota
(former Verrucomicrobia), Mycoplasmatota (former Tenericutes), Pseudomonadota (former Proteobacteria) and Fusobacteriota (former Fusobacteria).

## Slide 7
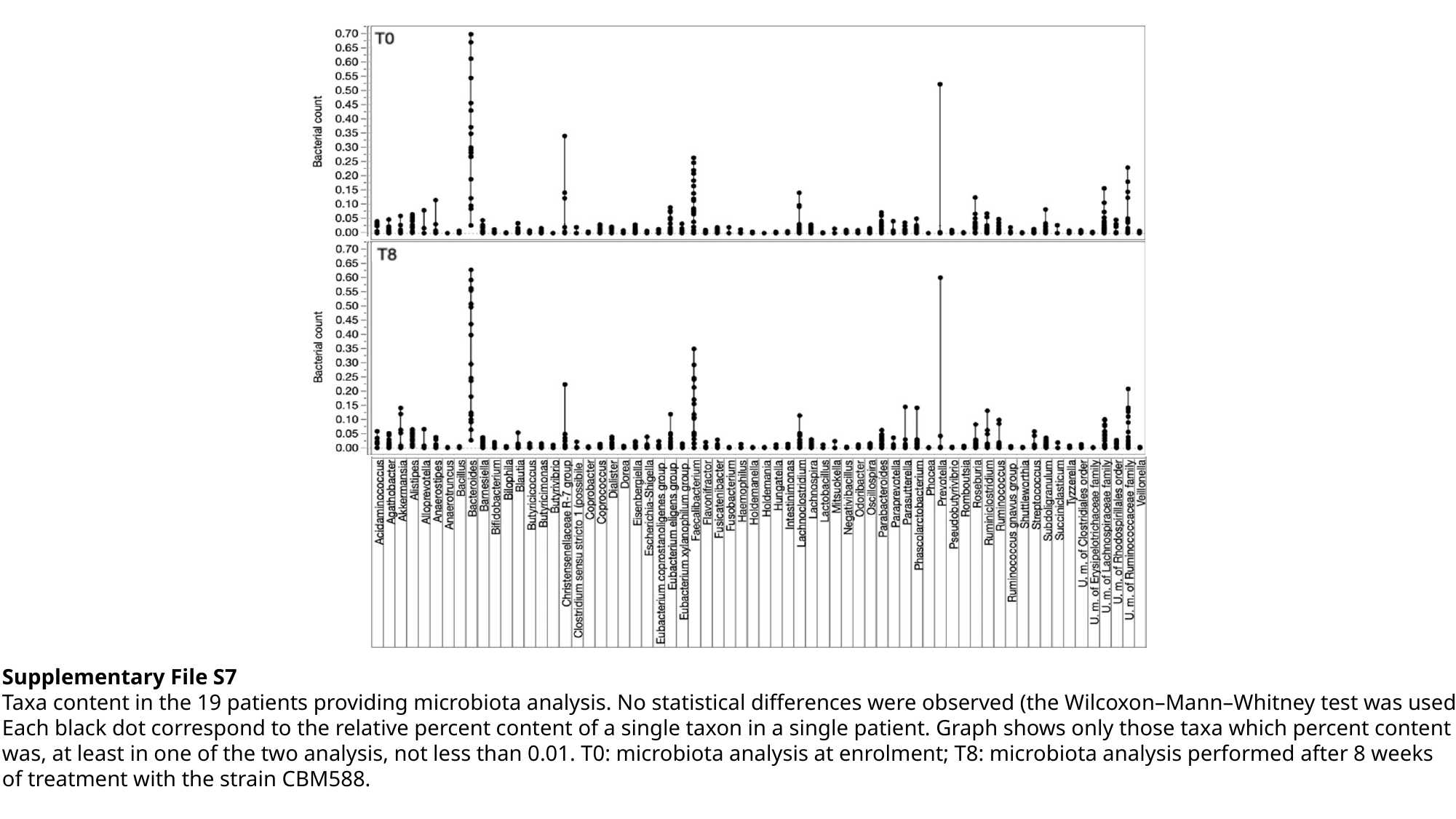

Supplementary File S7
Taxa content in the 19 patients providing microbiota analysis. No statistical differences were observed (the Wilcoxon–Mann–Whitney test was used).
Each black dot correspond to the relative percent content of a single taxon in a single patient. Graph shows only those taxa which percent content
was, at least in one of the two analysis, not less than 0.01. T0: microbiota analysis at enrolment; T8: microbiota analysis performed after 8 weeks
of treatment with the strain CBM588.

## Slide 8
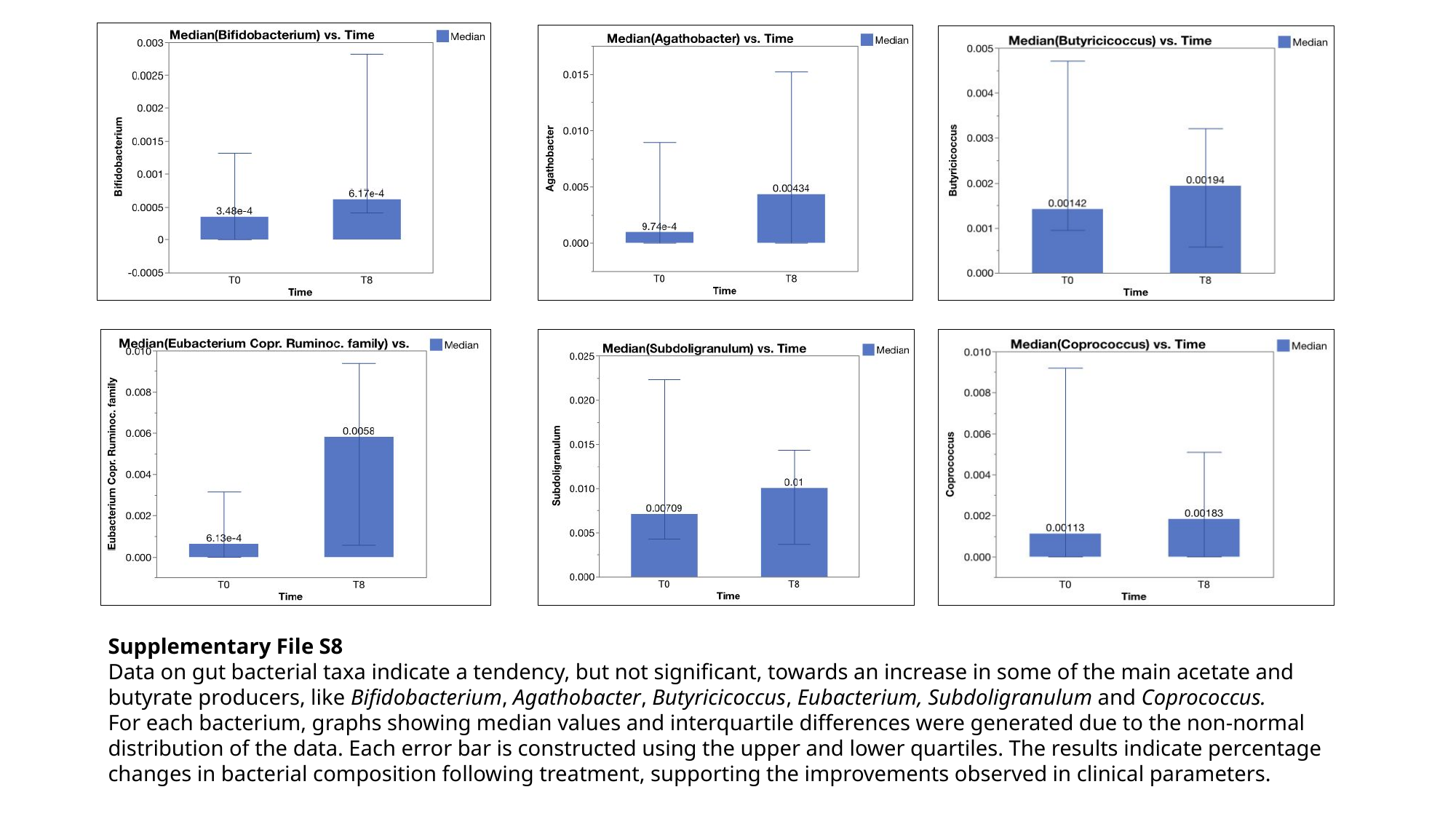

Supplementary File S8
Data on gut bacterial taxa indicate a tendency, but not significant, towards an increase in some of the main acetate and butyrate producers, like Bifidobacterium, Agathobacter, Butyricicoccus, Eubacterium, Subdoligranulum and Coprococcus.
For each bacterium, graphs showing median values and interquartile differences were generated due to the non-normal distribution of the data. Each error bar is constructed using the upper and lower quartiles. The results indicate percentage changes in bacterial composition following treatment, supporting the improvements observed in clinical parameters.

## Slide 9
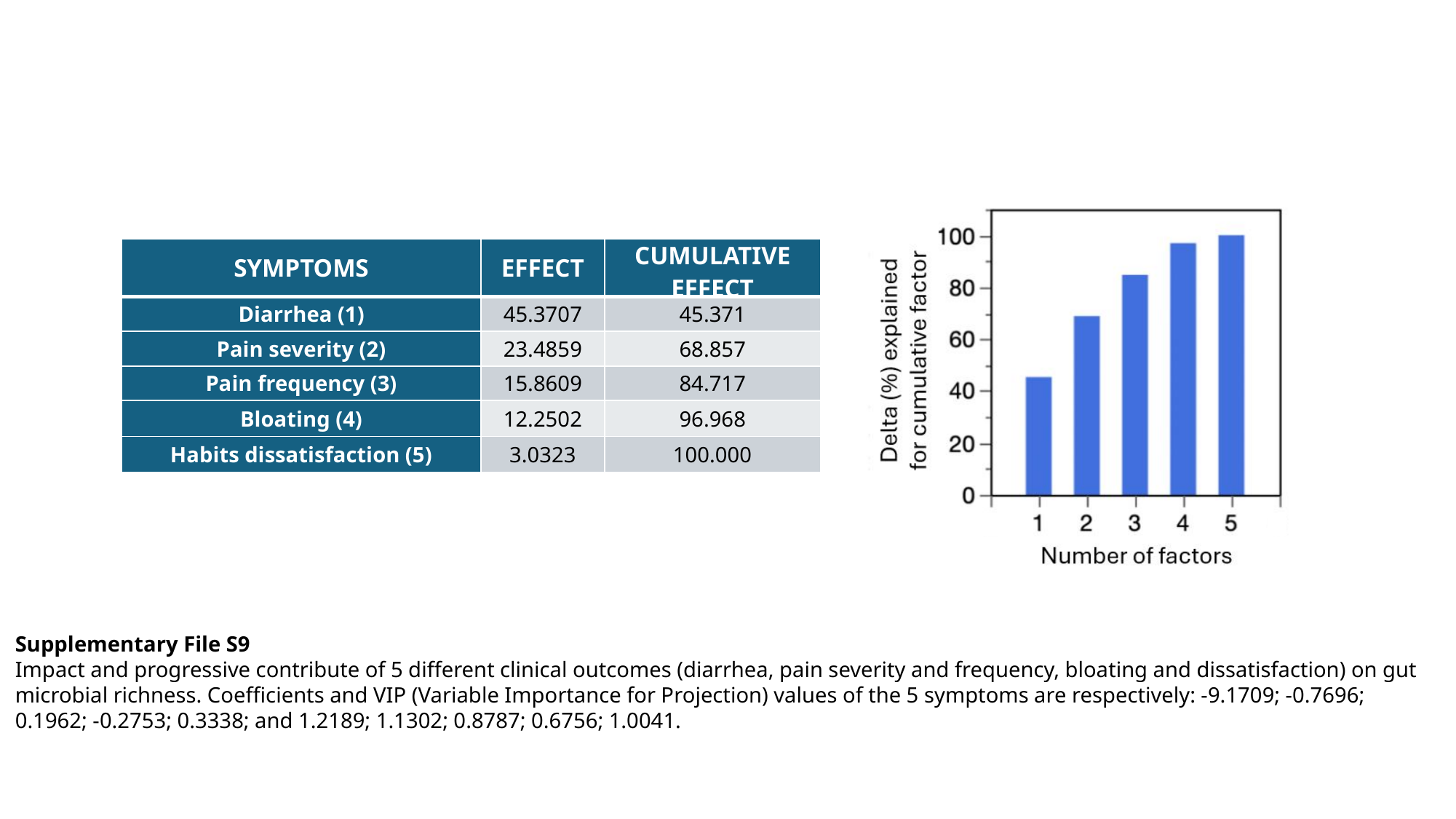

| SYMPTOMS | EFFECT | CUMULATIVE EFFECT |
| --- | --- | --- |
| Diarrhea (1) | 45.3707 | 45.371 |
| Pain severity (2) | 23.4859 | 68.857 |
| Pain frequency (3) | 15.8609 | 84.717 |
| Bloating (4) | 12.2502 | 96.968 |
| Habits dissatisfaction (5) | 3.0323 | 100.000 |
Supplementary File S9
Impact and progressive contribute of 5 different clinical outcomes (diarrhea, pain severity and frequency, bloating and dissatisfaction) on gut
microbial richness. Coefficients and VIP (Variable Importance for Projection) values of the 5 symptoms are respectively: -9.1709; -0.7696;
0.1962; -0.2753; 0.3338; and 1.2189; 1.1302; 0.8787; 0.6756; 1.0041.

## Slide 10
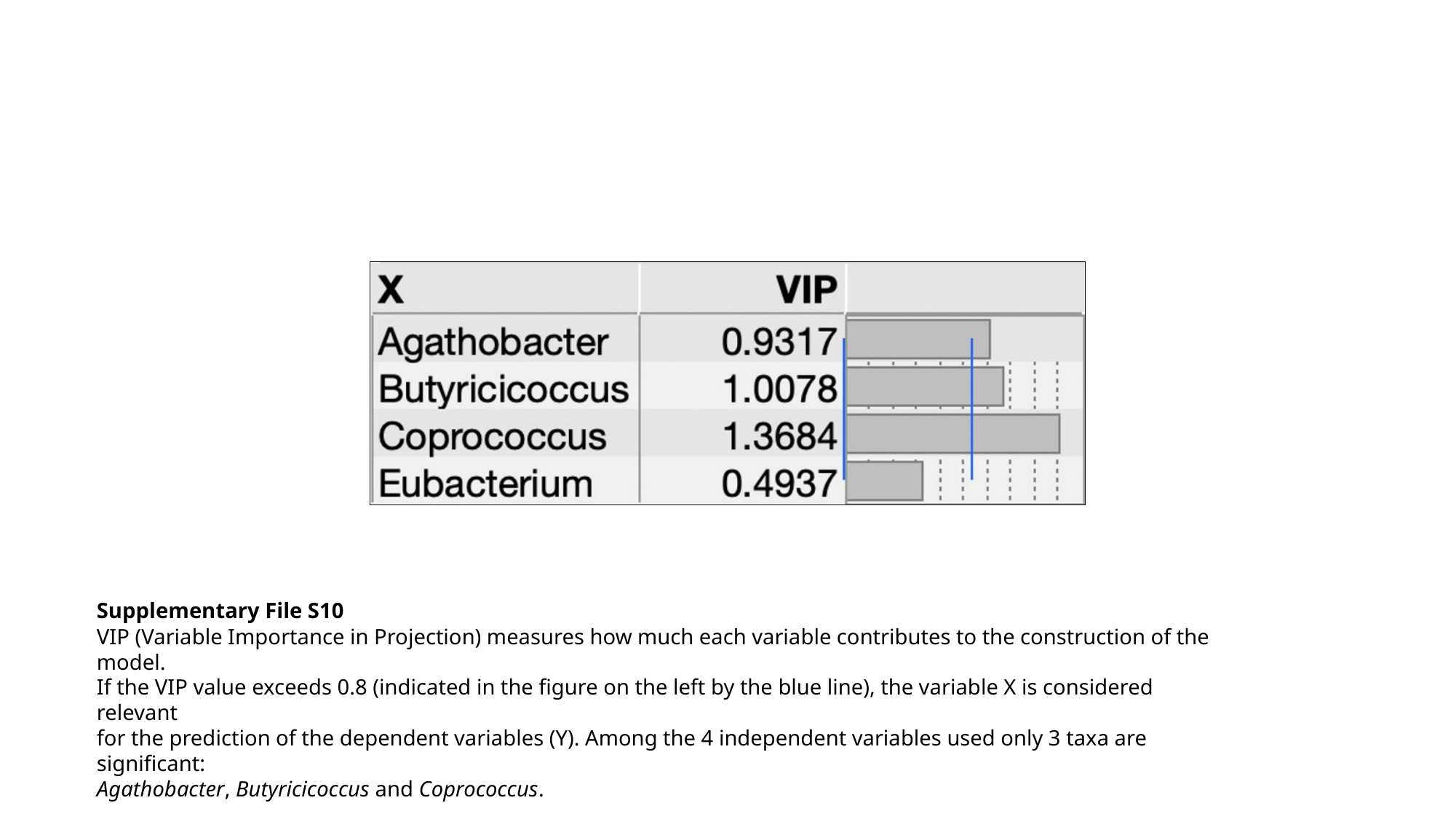

Supplementary File S10
VIP (Variable Importance in Projection) measures how much each variable contributes to the construction of the model.
If the VIP value exceeds 0.8 (indicated in the figure on the left by the blue line), the variable X is considered relevant
for the prediction of the dependent variables (Y). Among the 4 independent variables used only 3 taxa are significant:
Agathobacter, Butyricicoccus and Coprococcus.

## Slide 11
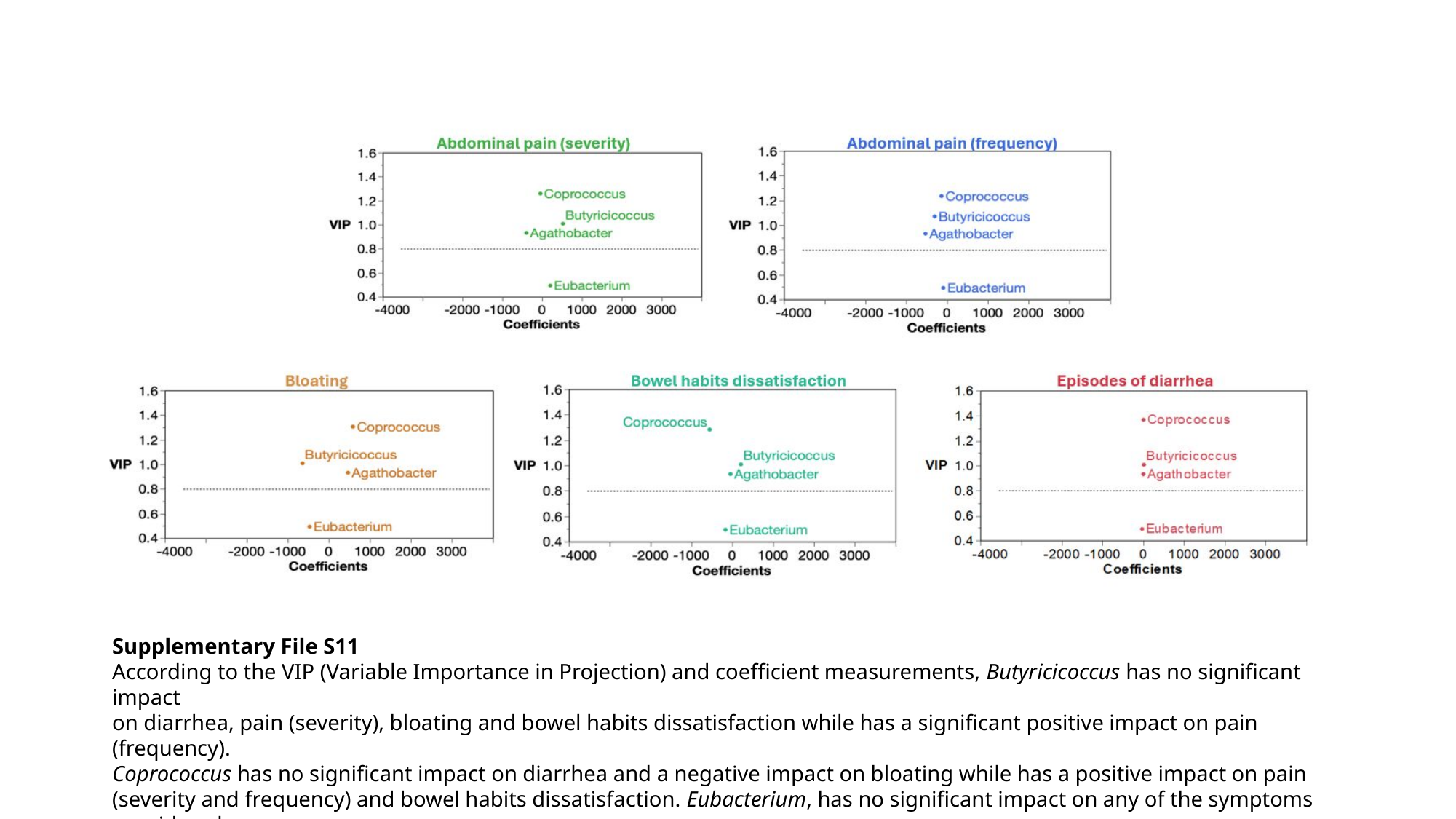

Supplementary File S11
According to the VIP (Variable Importance in Projection) and coefficient measurements, Butyricicoccus has no significant impact
on diarrhea, pain (severity), bloating and bowel habits dissatisfaction while has a significant positive impact on pain (frequency).
Coprococcus has no significant impact on diarrhea and a negative impact on bloating while has a positive impact on pain
(severity and frequency) and bowel habits dissatisfaction. Eubacterium, has no significant impact on any of the symptoms considered.

## Slide 12
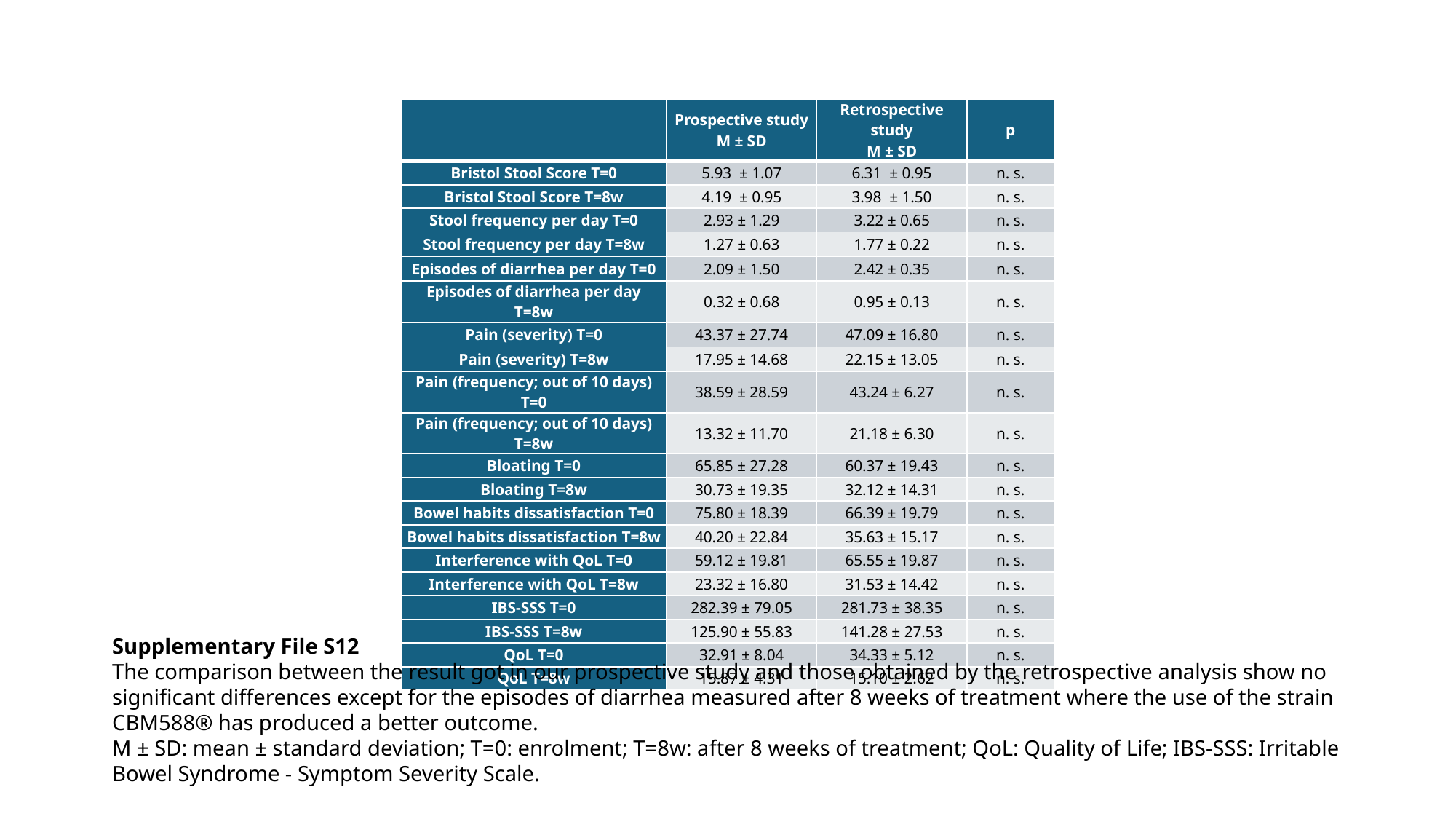

| | Prospective study M ± SD | Retrospective study M ± SD | p |
| --- | --- | --- | --- |
| Bristol Stool Score T=0 | 5.93 ± 1.07 | 6.31 ± 0.95 | n. s. |
| Bristol Stool Score T=8w | 4.19 ± 0.95 | 3.98 ± 1.50 | n. s. |
| Stool frequency per day T=0 | 2.93 ± 1.29 | 3.22 ± 0.65 | n. s. |
| Stool frequency per day T=8w | 1.27 ± 0.63 | 1.77 ± 0.22 | n. s. |
| Episodes of diarrhea per day T=0 | 2.09 ± 1.50 | 2.42 ± 0.35 | n. s. |
| Episodes of diarrhea per day T=8w | 0.32 ± 0.68 | 0.95 ± 0.13 | n. s. |
| Pain (severity) T=0 | 43.37 ± 27.74 | 47.09 ± 16.80 | n. s. |
| Pain (severity) T=8w | 17.95 ± 14.68 | 22.15 ± 13.05 | n. s. |
| Pain (frequency; out of 10 days) T=0 | 38.59 ± 28.59 | 43.24 ± 6.27 | n. s. |
| Pain (frequency; out of 10 days) T=8w | 13.32 ± 11.70 | 21.18 ± 6.30 | n. s. |
| Bloating T=0 | 65.85 ± 27.28 | 60.37 ± 19.43 | n. s. |
| Bloating T=8w | 30.73 ± 19.35 | 32.12 ± 14.31 | n. s. |
| Bowel habits dissatisfaction T=0 | 75.80 ± 18.39 | 66.39 ± 19.79 | n. s. |
| Bowel habits dissatisfaction T=8w | 40.20 ± 22.84 | 35.63 ± 15.17 | n. s. |
| Interference with QoL T=0 | 59.12 ± 19.81 | 65.55 ± 19.87 | n. s. |
| Interference with QoL T=8w | 23.32 ± 16.80 | 31.53 ± 14.42 | n. s. |
| IBS-SSS T=0 | 282.39 ± 79.05 | 281.73 ± 38.35 | n. s. |
| IBS-SSS T=8w | 125.90 ± 55.83 | 141.28 ± 27.53 | n. s. |
| QoL T=0 | 32.91 ± 8.04 | 34.33 ± 5.12 | n. s. |
| QoL T=8w | 15.87 ± 4.31 | 15.10 ± 2.62 | n. s. |
Supplementary File S12
The comparison between the result got in our prospective study and those obtained by the retrospective analysis show no significant differences except for the episodes of diarrhea measured after 8 weeks of treatment where the use of the strain CBM588® has produced a better outcome.
M ± SD: mean ± standard deviation; T=0: enrolment; T=8w: after 8 weeks of treatment; QoL: Quality of Life; IBS-SSS: Irritable Bowel Syndrome - Symptom Severity Scale.
